# Climate change projection using statistical downscaling model over southern coastal Iran

**DOI:** 10.1016/j.heliyon.2024.e29416

**Published:** 2024-04-12

**Authors:** Sorour Esfandeh, Afshin Danehkar, Abdolrassoul Salmanmahiny, Hassan Alipour, Majid Kazemzadeh, Marina Viorela Marcu, Seyed Mohammad Moein Sadeghi

**Affiliations:** a Department of Environmental Science and Engineering, Faculty of Natural Resources, University of Tehran, Karaj, Iran; b Department of Environmental Science, Faculty of Fisheries and Environmental Sciences, Gorgan University of Agricultural Sciences and Natural Resources, Gorgan, Iran; c Department of Arid and Mountain Reclamation Engineering, Faculty of Natural Resources, University of Tehran, Karaj, Iran; d Faculty of Natural Resources and Environment, Ferdowsi University of Mashhad, Mashhad, Iran; e Department of Forest Engineering, Forest Management Planning and Terrestrial Measurements, Faculty of Silviculture and Forest Engineering, Transilvania University of Brasov, Şirul Beethoven 1, 500123, Brasov, Romania; f School of Forest, Fisheries, and Geomatics Sciences University of Florida, Gainesville, FL, USA

**Keywords:** Iran, Climate change projection, Southern coastal Iran, CMIP5, CMIP6, Mean daily temperature, Total daily rainfall

## Abstract

Iran is highly vulnerable to climate change, particularly evident in shifting precipitation and temperature patterns, especially in its southern coastal region. With these changing climate conditions, there is an urgent need for practical and adaptive management of water resources and energy supply to address the challenges posed by future climate change. Over the next two to three decades, the effects of climate change, such as precipitation and temperature, are expected to worsen, posing greater risks to water resources, agriculture, and infrastructure stability. Therefore, this study aims to evaluate the alterations in mean daily temperature (T_mean_) and total daily rainfall (rrr24) utilizing climate change scenarios from both phases 5 and 6 of the Coupled Model Inter-comparison Project (CMIP5 and CMIP6, respectively) in the southern coastal regions of Iran (Hormozgan province), specifically north of the Strait of Hormuz. The predictions were generated using the Statistical Downscaling Model (SDSM) and National Centre for Environmental Prediction (NCEP) predictors, incorporating climate change scenarios from CMIP5 with Representative Concentration Pathways (RCPs) 2.6, 4.5, and 8.5 and CMIP6 with Shared Socioeconomic Pathways (SSPs) 1, 2, and 5. The analysis was conducted for three distinct time periods: the early 21st century (2021–2045), middle 21st century (2046–2071), and late 21st century (2071–2095). The results indicated that the CMIP5 model outperformed the CMIP6 model in simulating and predicting T_mean_ and rrr24. In addition, a significant increase in T_mean_ was observed across all the scenarios and time periods, with the most pronounced trend occurring in the middle and late 21st century future periods. This increase was already evident during the base period of 2021–2045 across all scenarios. Moreover, the fluctuations in precipitation throughout the region and across all scenarios were significant in the three examined future periods. The results indicated that among CMIP5 scenarios, RCP8.5 had highest changes of T_mean_ (+1.22 °C) in Bandar Lengeh station in 2071–2095 period. The lowest change magnitude of T_mean_ among CMIP5 scenarios was found in RCP4.5 (−1.94 °C) in Ch station in 2046–2070 period. The results indicated that among CMIP5 scenarios, RCP8.5 had highest changes of rrr24 (+150.2 mm) in Chabahar station in 2071–2095 period. The lowest change magnitude of rrr24 among CMIP5 scenarios was found in RCP8.5 (−25.8 mm) in Bandar Abbas station in 2046–2070 period. In conclusion, the study reveals that the coastal area of Hormozgan province will experience rising temperatures and changing rainfall patterns in the future. These changes may lead to challenges such as increased water and energy consumption, heightened risks of droughts or floods, and potential damage to agriculture and infrastructure. These findings offer valuable insights for implementing local mitigation policies and strategies and adapting to emerging climate changes in Hormozgan's coastal areas. For example, utilizing water harvesting technologies, implementing watershed management practices, and adopting new irrigation systems can address challenges like water consumption, agricultural impacts, and infrastructure vulnerability. Future research should accurately assess the effect of these changes in precipitation and temperature on water resources, forest ecosystems, agriculture, and other infrastructures in the study area to implement effective management measures.

## 1 Introduction

Climate change refers to long-term shifts and changes in temperatures and precipitation patterns, mainly caused by human activities, especially the burning of fossil fuels. The global mean surface temperature has increased approximately 1 °C between 1850 and 2020, leading to significant warming over both land and ocean [1]. Strong evidence from climate change research implicates human-induced greenhouse gas (GHG) emissions, with concentrations primarily of carbon dioxide (CO_2_) and methane (CH_4_), reaching historically high mean annual values during 2010–2019 [[2], [3], [4], [5]]. The consequences of global warming are widespread and observable worldwide. In particular, global warming has disturbed global atmospheric circulation and water cycle components, including evaporation and precipitation [6,7]. The increasing mean global land precipitation, sea-level rise, retreat of glaciers, and poleward shift of mid-latitude storms are among the most prominent environmental impacts of global warming and associated climate change [8,9]. In addition, these changes pose numerous challenges in the realms of social, economic, and safety considerations [10], which are more severe in human-dominated flood-prone areas and extreme climate landscapes [11,12]. Additionally, dry regions are confronting complex hydroclimatic hazards, notably drought, with severe repercussions on human activities. These consequences encompass the complete failure of agricultural endeavors, human migration, and famine [13,14].

Coastal countries stand out as the most at-risk regions on Earth due to the magnitude of climate change [15]. Floods alone affected 2.3 billion people worldwide from 1998 to 2015 and the majority (95%) of those people live in Asia [16]. Coastal plains are attracting a significant share of the global population and development activities worldwide [17]. Despite covering only 2% of the total land surface, it is estimated that coastal migration will drive the emergence of future megacities in coastal zones worldwide [18]. Nevertheless, coastal areas are exceptionally vulnerable to global warming and climate change, with an expected increase in inundation and episodic flooding tides [[19], [20], [21]]. Therefore, it is imperative to recognize the importance of restraining global warming and maintaining a constant vigilance over its attributes in coastal regions. This awareness highlights the imperative to build more resilient infrastructure and communities in coastal regions.

In recent decades, considerable scientific endeavors have resulted in the creation of multiple models designed to predict future climate conditions based on various scenarios. One notable contribution to advancing our understanding of climate science has been the Coupled Model Intercomparison Project (CMIP), which has been active since the 1990s [8]. The CMIP has significantly advanced our understanding of climate science. The studies indicated that global warming is causing and affecting climate changes based on the Coupled Model Intercomparison Project Phase 5 (CMIP5) and Coupled Model Intercomparison Project Phase 6 (CMIP6) climate reports. Compared to other procedures such as the multi-model ensemble (MME), the CMIP6 and CMIP5 models exhibited better performance in the past studies. To study the climate change projection, with the release of the sixth report (CMIP6), the desire to examine the performance of this report compared to the fifth report (CMIP5) has increased among researchers [66]. The CMIP has enabled the use of Representative Concentration Pathway (RCP) emission scenarios. These scenarios were first introduced in CMIP5 [22] and have been continued in CMIP6 [23] to provide more precise insights into climate responses and mechanisms. CMIP6 models have been enhanced with higher spatial resolution, approximately 100 km. Furthermore, CMIP6 models now incorporate the new Shared Socioeconomic Pathway (SSP)/RCP-based emission scenarios. Also, the sixth phase of CMIP (CMIP6) tries to address novel scientific inquiries within the realm of climate change. This effort has garnered participation from 33 research institutions globally, signifying its widespread engagement [23]. In contrast to CMIP5, CMIP6 revealed enhanced oceanic and atmospheric resolution. In addition, CMIP6 incorporates intricate and novel mechanisms, encompassing more elaborate land surface phenomena, permafrost behaviors, ice field dynamics, and other complex processes [24].

In general, CMIP6 global climate models have been widely used to project twenty-first-century temperatures and assess potential climate change risks. However, these models exhibit a transient climate response spanning from 1.2 to 2.8 °C and an equilibrium climate sensitivity ranging from 1.8 to 5.7 °C. Consequently, significant disparities exist in the projected climatic effects attributed to the rise in atmospheric CO_2_ levels caused by human activities. In addition, mounting evidence suggests that numerous global climate models are producing results that are excessively warm, thereby compromising their reliability in guiding policies related to future climate shifts [25,26]. Furthermore, a significant concern lies with clouds [27]. As the atmosphere warms, a transition occurs within the cloud population, shifting from ice and mixed-phase clouds (referred to as 'cold') to liquid clouds (referred to as 'warm'). Warm clouds have higher reflectivity and longer lifespans, leading to a reduction in the solar energy absorbed by the Earth and resulting in a negative radiative feedback. Notably, this cooling feedback is less pronounced in CMIP6 compared to CMIP5, thereby contributing to intensified greenhouse warming [28,29]. A review of the existing literature indicates that CMIP6 still encounters challenges in accurately replicating climate change and its inherent variability [30,31].

Despite the advancements in CMIPs, they still have limitations, primarily due to their coarse horizontal resolutions. To obtain high resolution, several models for downscaling, both statistical and dynamic, have been created. Downscaling models aim to provide detailed and localized climate information by refining CMIP model outputs. In summary, the combination of CMIPs and downscaling models has greatly enhanced our understanding of future climate projections [32,33], but ongoing efforts continue to improve the accuracy and resolution of these models. Kazemzadeh et al. [34] reported nonlinear changes and spatiotemporal trends in air temperature over Iran during 1978–2017. Thus, it is necessary to study climate changes projections over the country to develop the effective management strategies, especially in southern coasts of Iran. This study aims to assess changes in mean daily temperature and total daily rainfall in three future time periods of 2021–2045, 2046–2071, and 2071–2095 over the southern coastal region of Iran using climate change scenarios from both CMIP5 and CMIP6 models to contribute to the current knowledge. Moreover, in order to find past and future changes in precipitation and temperature data on a regional scale, trend analysis is also performed. The study focuses on the southern coasts of Iran, north of the Strait of Hormuz, where climate change has had a significant impact on human activity and the viability of the region's ecosystem.

## 2 Materials and methods

### 2.1 Study area descriptions

**Fig. 1:**
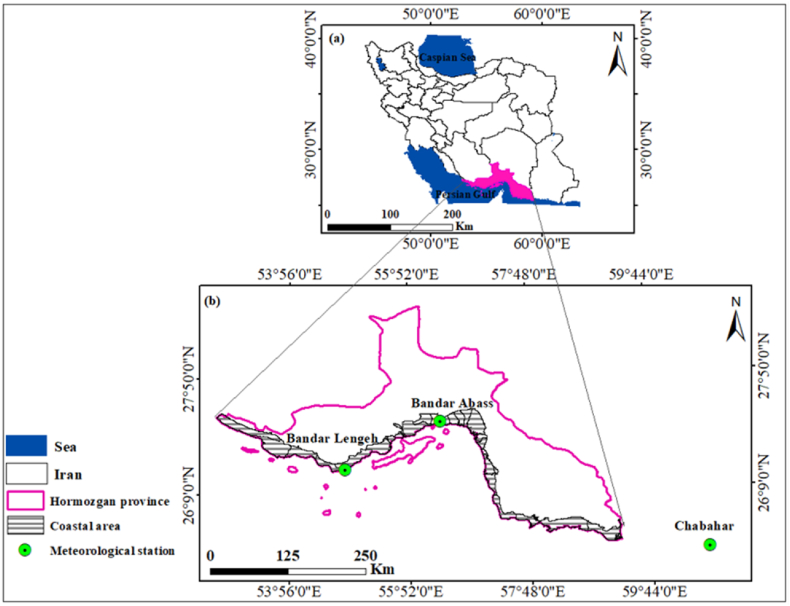
Map of: (a) provinces of Iran, (b) the study area Hormozgan province with its southern coast.

The research was carried out in a coastal area located in Hormozgan province, Iran, specifically within the coordinates of 25°–28° N and 53°–59° E (Fig. 1a and b). The study area covers approximately 10,500 km^2^. The coastal region, extending approximately 900 km in the south, is characterized by its proximity to the warm waters of the Persian Gulf and the Oman Sea. This coastal area holds strategic importance for Iran. Hormozgan province which is renowned for its hot and arid climate, characterized by low annual precipitation levels averaging 168 mm [35]. The region experiences a notable absence of rainfall for approximately 9 months of the year, with most precipitation events occurring only once or twice. The coastal climate experiences high temperatures and humidity during the summer, with occasional temperatures surpassing 52 °C. The average annual temperature in this region is around 27 °C.

### 2.2 Data description

The mean air temperature (T_mean_) and total daily rainfall (rrr24) data were collected from the Iranian Meteorological Organization for three stations: Bandar Abbas and Bandar Lengeh in Hormozgan province, and Chabahar in Sistan and Balochestan province. These data cover the period from 1966 to 2020. Since there are no other reliable meteorological stations with long-term datasets in the eastern part of the coastal region, the data from the Chabahar station were used to represent that area for climatic prediction and evaluation purposes. The National Center for Environmental (NCEP) reanalysis dataset [36] was collected and used to calibrate and validate the models. In addition, in order to generate scenarios, two groups of 26 large-scale predictors obtained from the Canadian Earth System Model version 2 (CanESM2) in the CMIP5 experiment (RCP2.6, 4.5, and 8.5 scenarios) and the Canadian Earth System Model version 5 (CanESM5) in the CMIP6 experiment (SSP126, SSP245, and SSP585 scenarios) were selected. These scenarios were used to project climate conditions for early 21st century (2021–2045), middle 21st century (2046–2070), and late 21st century (2071–2095).

### 2.3 Statistical downscaling model

At the local level, the Factual Downscaling Show, developed by Wilby et al. [37], plays a crucial role as a valuable tool in facilitating decision-making processes for assessing the impacts of climate change. After an extensive analysis conducted by Wilby and Dawson [38], by reviewing the initial version of 2001, it was concluded that the Statistical Downscaling Model (SDSM) has multiple applications. These applications encompass replicating historical weather patterns, forecasting future climate under different scenarios, infilling missing data, and reconstructing past climate information. The relevance of SDSM extends beyond conventional climatic variables such as temperature and precipitation to encompass exceptional factors like air quality and reference evapotranspiration, as well as climate extremes [39,40].

SDSM is an integrated approach that combines Multi Linear Regression (MLR) and the Stochastic Weather Generator (SWG). The SDSM framework incorporates the MLR technique to construct robust associations between the input variables (i.e., NCEP predictors) and output variables (i.e., predictands). SDSM has the ability to capture the inter-annual variability better than other statistical downscaling approaches, e.g. weather generators, and weather typing [39]. This process (the MLR technique) takes place during the selection of predictors (screen variables) and the subsequent calibration of the SDSM model. This leads to the estimation of regression parameters for the model. To closely match the observed data during the validation process, a maximum of 100 daily time series is generated using the calibration parameters derived from NCEP and General Circulation Model (GCM) predictors. It is common practice, supported by studies by Wilby et al. [37], and Chu et al. [41], to consider a set of twenty-time series as a benchmark for comparison. In SDSM various techniques such as partial correlations, correlation matrices, explained variance, *P*-values, histograms, and scatterplots can be employed to select a subset of appropriate predictors from a large pool of climatic indicators. It is important to consider multicollinearity, which can lead to misleading conclusions, during the selection of predictors. SDSM offers two optimization methods: Ordinary Least Squares (OLS) and Double Simplex (DS). OLS is a computationally faster method compared to Double Simplex and yields comparable outcomes [42,43].

SDSM employs two types of sub-models, namely unrestricted and conditional, based on the nature of the predictands. The unrestricted sub-model is applied to independent variables like T_mean_, whereas the conditional sub-model is employed for dependent variables such as rrr24 [38,44]. SDSM also provides calibrated parameters that are specifically designed for different time resolutions (i.e., monthly, seasonal, and annual scales). These calibrated parameters play a crucial role in improving the accuracy and reliability of the modeled climatic variables at different temporal scales. This capability allows for improved accuracy and reliability in modeling climatic variables across different time scales [38,44]. SDSM possesses the ability to normalize data by applying different transformations, ensuring its compatibility with regression techniques [45]. To construct SDSM, two types of daily time series are necessary: NCEP indicator and observed data [42]. Further technical information regarding this process can be found in the study conducted by Wilby et al. [43,46]. In current study, 70 % and 30 % of observed temperature and precipitation time series were used to calibration and validation process, respectively, during 1966–2005 for CMIP5 1979–2014 for CMIP6.

The scrutiny of substantial scale variables is a pivotal procedure in all forms of statistical downscaling, as evidenced by scholarly research [37,42]. Once comprehensive datasets have been obtained, the first step involves the identification of a diverse range of potential predictors for each specific climatic variable at every station. The foundation of all SDSM methodologies is the establishment of relationships between large-scale predictor variables vs. local predictands. These relationships serve as the foundation for downscaling and generating localized projections or estimates [37]. Moreover, these potential predictors must be competently modeled by GCMs and obtainable from numerous sources of GCMs [37,47]. The selection of suitable predictors is crucial for the success of the models. In this study, the identification of appropriate predictors is achieved through correlation analysis and the establishment of *P*-values. To do so, the most effective NCEP predictors for the T_mean_ and rrr24 at the meteorological stations are given in Table 1. These statistical techniques help determine the strength of the relationships between the predictors and the predictands and provide insights into their statistical significance. The same combination was also used by Huang et al. [42], Mahmood and Babel [43,47], and Phuong et al. [48] for both of CMIP5 and CMIP6 models.

**Table 1:** The most effective National Centre for Environmental Prediction (NCEP) predictors for the mean daily temperature (T_mean_) and total daily rainfall (rrr24) at the meteorological stations.

Model	T_mean_	rrr24
CMIP5	ncepmslpgl.dat	
	ncepp5_fgl.dat	ncepmslpgl.dat
	ncepp5_ugl.dat	ncepp5_fgl.dat
	ncepp500gl.dat	ncepp5_ugl.dat
	nceptempgl.dat	nceptempgl.dat
CMIP6	p1_v_day	prcp_day
	temp_day	s850_day

### 2.4 Model setup and execution

Giving a brief review of downscaling techniques, we presented the structure and operation of SDSM with respect to five steps: (1) preliminary screening of potential downscaling predictor variables; (2) assembly and calibration of SDSM; (3) synthesis of ensembles of current weather data using observed predictor variables; (4) generation of ensembles of future weather data using GCM-derived predictor variables; (5) diagnostic testing/analysis of observed data and climate change scenarios [37]. This paper concludes with an application of SDSM to climate change scenario generation for southern coastal Iran, comparing downscaled daily precipitation and temperature series for 2021–2045, 2046–2070 and 2071–2095.

### 2.5 Model performance evaluation

It is recommended to utilize measures based on absolute error/deviation. In this study, mean square error (MSE), root mean square error (RMSE), and median absolute deviation (MAD) were employed to evaluate the accuracy of the models. These metrics are considered more appropriate for assessing model performance in hydrological and environmental studies [10,[49], [50], [51]]. MSE is the squared mean of the difference between the observed values and predictable values. RMSE is the standard deviation of the errors which occur when a prediction is made on a dataset. This is the same as MSE but the root of the value is considered while determining the accuracy of the model. MAD is the absolute difference between the observed values and the values that are predicted. Therefore, for all model metrics, the lower the value is, the more accurate the simulation and prediction of the model will be. To calculate RMSE, MSE and MAD, T_mean_ and rrr24 time series for the study periods were used. In the following equations, y_i_ represents the observed value, ${\hat{y}}_{i}$ is the corresponding predicted value, and n represents the total number of observations.

(1) $$MSE={\frac{1}{n}\sum_{i=n}^{n}\left(y_{i}-{\hat{y}}_{i}\right)}^{2}$$

(2) $$RMSE=\sqrt{\frac{1}{n}\sum_{i=1}^{n}{\left(y_{i}-{\hat{y}}_{i}\right)}^{2}}$$

(3) $$MAD=\frac{1}{n}\sum_{i=n}^{n}|y_{i}-{\hat{y}}_{i}|$$

### 2.6 The innovative- Sen trend analysis (ITA)

In this study, the ITA method, developed by Sen [52], is employed to detect and analyze both overall and partial trends within the hydrometeorological time series being examined. The ITA method does not impose strict assumptions related to parametric tests. This characteristic enables a comprehensive assessment of trend patterns in the study by providing a more accurate understanding of the trends in the data [53]. This makes the ITA method more flexible and applicable in diverse data scenarios. The inspiration for this study stemmed from a well-conducted investigation that examined the potential trends across multiple time durations in global monthly temperature records [54]. In this study, the ITA method was utilized to analyze the trend patterns observed in the time series of T_mean_ and rrr24. Specifically, the focus was on examining the trend behavior between the reference period of 1980–2009 and the future periods of 2020–2045, 2046–2070, and 2071–2095. This analysis was conducted considering multiple scenarios. So to do, the construction of the ITA plot involves arranging the ordered sequence of the 1980–2009 along the x-axis, while the ordered sequence of future periods (i.e., 2020–2045, 2046–2070, and 2071–2095) are positioned along the y-axis. The next step is to draw the 1:1 (45°) straight line and ±10 % error lines on the Cartesian coordinate system. The 45° straight line divides the plot into two equal regions, serves as a reference line, while the upper triangular area represents increasing trends, and the lower triangular area represents decreasing trends. In addition, the 45° straight line indicates cases where no clear trend is observed. Furthermore, in cases where the scatter points in the plot deviate significantly from the 45° straight line, the trend slope is considered to be stronger. This deviation indicates a more pronounced trend in the data, indicating a clear direction of change over time [40,52].

### 2.7 Time series analysis

Time series analysis garnered the attention of numerous researchers in recent decades across diverse fields, including dendrochronology [55], forest economy [56], land management [57], ecohydrology [58], and water resources management [59]. The primary objective of conducting time series analysis on a phenomenon is to develop a statistical model using historical data, thereby enabling the prediction of future outcomes for that specific phenomenon [60]. To put it differently, time series analysis involves constructing a historical model to facilitate informed choices in the future. A sequence of data collected in a time limit forms a time series. These data reflect the changes that the phenomenon has undergone over time. Therefore, we can consider these values as a time-dependent vector. In this case, if X is a vector, the time series can be shown as follows; where t represents time and X is a random variable (Equation (4)). In this study, our aim is to enhance our understanding of the behavior and fluctuations of T_mean_ and rrr24 within the coastal strip of Hormozgan province during the historical period. Additionally, we seek to validate the modeled predictions of these two variables across various future time periods and scenarios. This validation is achieved through the utilization of time series analysis [61].

(4) x(t),t=0,1,2,…

## 3 Results and discussion

### 3.1 Model calibration and validation

For the calibration and validation of SDSM models, the observed T_mean_ and rrr24 datasets were utilized. In addition, the study incorporated relevant predictors that were derived from the NCEP reanalysis dataset. These predictors were carefully selected based on their appropriateness and were integrated into the analysis to enhance its validity and accuracy. The calibration period spanned from 1966 to 1995, while the validation period covered 1996 to 2005. These periods were specifically chosen for the CMIP5 experiment. Furthermore, for the CMIP6 experiment, the dataset encompassed the years 1979–2014, which were employed to calibrate and validate the SDSM model.

**Fig. 2:**
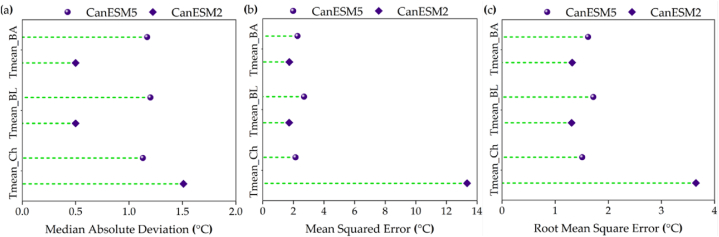
The model performance metrics in ^0^C during validation of models for daily mean air temperature (T_mean_) time series at three meteorological stations namely Bandar Abbas (BA), Bandar Lengeh (BL) and Chabahar (Ch) stations for: a) Median absolute deviation, b) Mean square error, and c) Root mean square error.

**Fig. 3:**
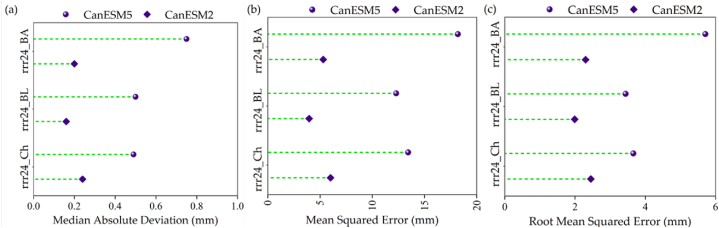
The model performance metrics in mm during validation of models for daily total rainfall (rrr24) time series at three meteorological stations namely Bandar Abbas (BA), Bandar Lengeh (BL) and Chabahar (Ch) stations for: a) Median absolute deviation, b) Mean square error, and c) Root mean square error.

Fig. 2, Fig. 3 display the validation results of the CanESM2 (CMIP5) and CanESM5 (CMIP6) models, respectively. Fig. 2, Fig. 3 also provide visual representations of how well the models perform in terms of these statistical metrics. The three-performance metrics of the two models indicated that the CanESM2 model has better estimated T_mean_ in the Bandar Lengeh and Bandar Abbas stations (Fig. 2a–c). While the accuracy of T_mean_ estimation by CanESM5 is relatively the same in all three stations (Fig. 2a–c). However, for the rrr24 in all three meteorological stations, the CanESM2 model performed better than the CanESM5 model (Fig. 3a–c). The values of MAD for T_mean_ and rrr24 vary approximately 0.5–1.5 °C and 0.16–0.75 mm, respectively in the two models (Fig. 2, Fig. 3a). It is visible that the values of MSE are between 1.73 and 13.35 °C, and 3.96–18.2 mm for T_mean_ and rrr24, respectively in both models (Fig. 2, Fig. 3b). Therefore, with a very small difference, the T_mean_ is better estimated with this statistic. Also, RMSE values were between 1.31 and 3.65 °C and 1.99–5.71 mm for T_mean_ and rrr24, respectively in the two models (Fig. 2, Fig. 3c). Considering the small difference of the error range in MAD (Fig. 2, Fig. 3a), in general, the T_mean_ is better estimated for all three stations. The reason for this is the climatic characteristics and the lack of rainfall in the region. In addition, the validation results suggest that the dimensional statistics obtained from the models fall within an acceptable range when considering the daily time scale. This indicates that the models are able to capture the observed data reasonably well in terms of their magnitude, demonstrating their suitability for daily-scale analyses. Overall, the evaluation of model accuracy revealed that the CMIP5 model outperformed the CMIP6 model in simulating and predicting T_mean_ and rrr24, across all time periods and scenarios, this confirms the weakness of the CMIP6 model. Also, Hamed et al. [62] reported the CMIP6 GCMs did not appear to perform better than the older CMIP5 in some parts of Asia unlike other parts of the globe. Srivastava et al. [63] found that the multi-model medians of CMIP6 and CMIP5 exhibit similar biases in climatology and variability in contiguous US (CONUS) regions. Zamani et al. [64] reported that the precipitations simulated by the ensembles of CMIP6 and CMIP5 models were different in Northeastern Iran. Unlike current results, they suggested that CMIP6 outperformed CMIP5 in projecting precipitation. Whereas CMIP5 underestimated it in 77 % of the stations, CMIP6 overestimated precipitation in 70 % of the stations. CMIP5 models revealed better performance in 70 % of the stations only in autumn. This outcome aligns with findings from previous research [63,64].

### 3.2 Future climate projections

**Fig. 4:**
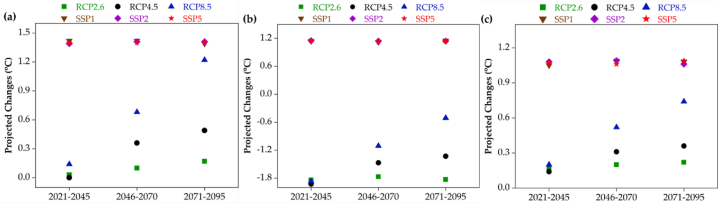
Projected changes in annual average temperature for the future periods under RCP2.6, RCP4.5 and RCP8.5 scenarios (CanESM2) and SSP1, SSP2, and SSP5 scenarios (CanESM5) at three meteorological stations namely: a) Bandar Lengeh (BL), b) Chabahar, and c) Bandar Abbas meteorological stations.

**Fig. 5:**
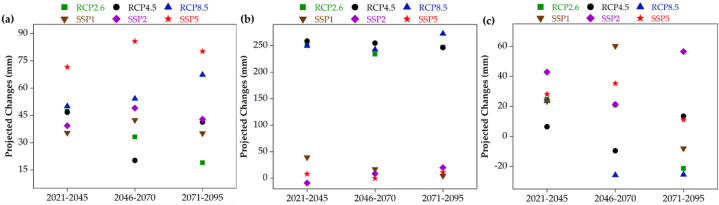
Projected changes in annual average rainfall for the future periods under RCP2.6, RCP4.5 and RCP8.5 scenarios (CanESM2) and SSP1, SSP2, and SSP5 scenarios (CanESM5) at three meteorological stations namely: a) Bandar Lengeh (BL), b) Chabahar, and c) Bandar Abbas meteorological stations.

Fig. 4, Fig. 5 display the projected changes in T_mean_ and rrr24, respectively. The calculations involve delta statistics, including both absolute and percentage differences, for the periods 2021–2045, 2046–2070, and 2071–2095 compared to the reference period, under multiple scenarios: CanESM2 RCP2.6, RCP4.5, RCP8.5, and CanESM5 SSP1, SSP2, SSP5. Based on Fig. 4, it can be observed that across all periods and scenarios, there is an increase in T_mean_ in the coastal strip of Hormozgan province, particularly in the Bandar Lengeh (Fig. 4a) and Bandar Abbas stations (Fig. 4c). The only exception is seen at the Chabahar station (eastern area) during the 2021–2045 period, as depicted in the RCPs. During the early 21st century period (2021–2045), there is an observed increase in T_mean_ across all scenarios, with a more significant rise in the SSPs. This increase becomes even more pronounced in the long-term future periods (2046–2070 and 2071–2095). Specifically, in the Bandar Lengeh station, the T_mean_ changes from 2021 to 2095 range from 0 to 1.2 °C under the RCPs. The lowest change is associated with RCP4.5 in the 2021–2045 period, while the highest change is related to RCP8.5 in the 2071–2095 period. However, in the SSPs, there will be an approximate T_mean_ increase of 1.5 °C in all periods. In the Bandar Abbas station (central part), the T_mean_ changes from 2021 to 2095 range from 0.1 to 0.7 °C under the RCPs. The lowest change is observed in RCP4.5 during the 2021–2045 period, while the highest change is seen in RCP8.5 during the 2071–2095 period. Similarly, in the SSPs, there will be an approximate T_mean_ increase of about 1 °C in all periods. T_mean_ changes in Chabahar station (eastern part) from 2021 to 2095 in the RCPs will be around 1.2 °C. But in the SSPs in all periods, we will face an increase in the T_mean_ of about 1.4 °C. Therefore, we will witness the most T_mean_ changes in the SSPs in Chabahar station. Also in the RCPs, the most changes in all parts of the region (all stations) are related to the RCP8.5. Also, in the RCPs, the least changes in all parts of the region (all stations) are related to the RCP2.6. In addition, compared to 2021–2045 period, the RCP8.5 with 1.2 °C in 2071–2095 is responsible for the most changes in the T_mean_ in the region (Fig. 4). As shown in Fig. 5, fluctuations of precipitation changes in the entire region and in all scenarios in the three time periods examined in the future are very high and evident. Fluctuations of precipitation changes in Bandar Lengeh station (western part) from 2021 to 2095 in the RCPs and SSPs are 19–67 mm and 35–85 mm, respectively. The lowest amount of rrr24 (−25.7 mm) is found for RCP8.5 in 2046–2070 period in Bandar Abbas station while the highest amount of rrr24 (272.5 mm) is related to RCP8.5 for 2071–2095 period in Chabahar station. For the SSPs, the lowest amount of rrr24 (−9.1 mm) is related to SSP2 for 2021–2045 period in Chabahar station whereas the highest amount of rrr24 (85.7 mm) is detected to SSP for 2046–2070 period in Bandar Lengeh station. The amount of rrr24 under the all scenarios and periods will be increased in Bandar Lengeh station, while it in some periods of Bandar Abbas station will be decreased. But the amount of rrr24 in Chabahar like Bandar Lengeh will be increased in the all of the scenarios and periods except SSP2 in 2021–2045. The fluctuations of precipitation changes in the eastern part of the region, specifically at the Chabahar station, is similar to the Bandar Lengeh station in the western part across all scenarios and throughout the investigated period. In addition, the intensity of increases under RCPs in Chabahar station will be higher than other stations. The climatic characteristics of the region, combined with the anticipated future climate changes, contribute to the fluctuation of high rainfall from low to high and vice versa (Fig. 5).

**Fig. 6:**
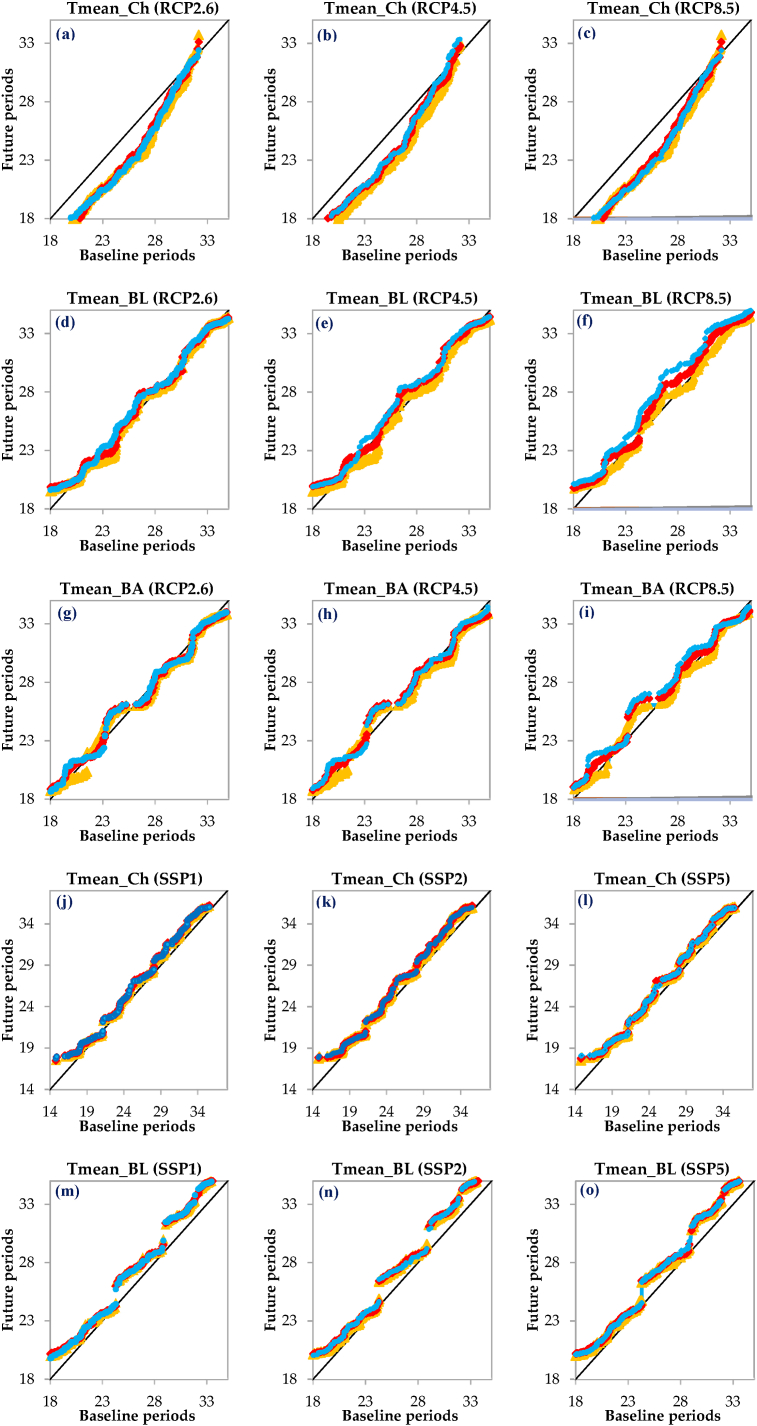

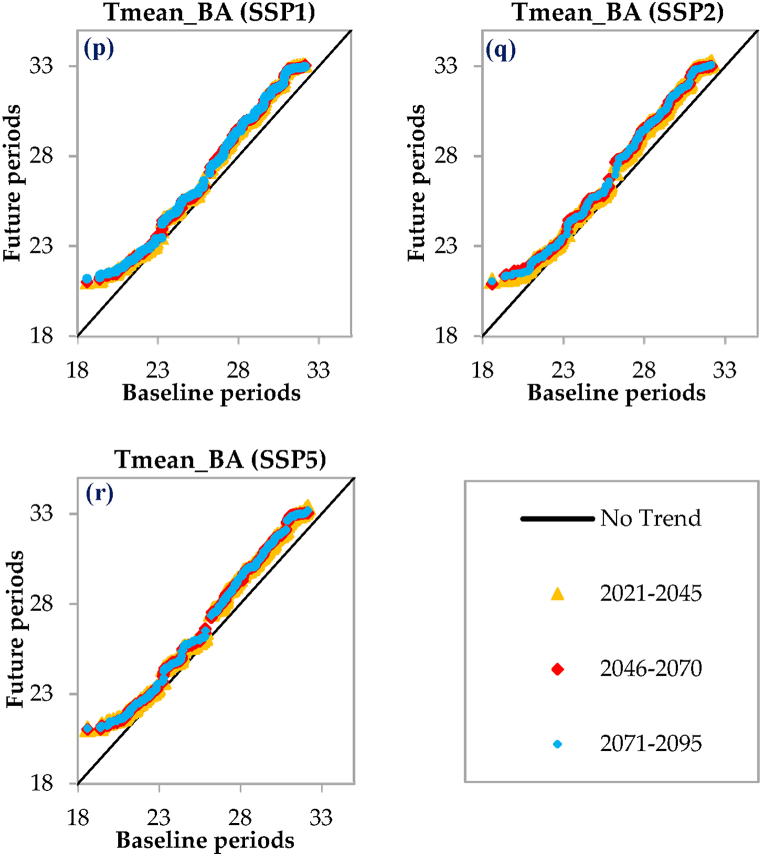
Innovative Trend Analysis (ITA) plots comparing the 2021–2045, 2046–2070, and 2071–2095 periods to the 1980–2009 across various scenarios. T_mean_ denotes the mean air temperature, while Ch, BL, and BA represent the Chabahar, Bandar Lengeh, and Bandar Abbas stations, respectively.

As shown in Fig. 6, the changes in T_mean_ for all three time periods and across the investigated scenarios exhibit a very similar behavior, indicating that there will not be a significant change in the T_mean_ in the flat areas under different scenarios. Nevertheless, upon examining the specifics at the Chabahar station located in the eastern part of the region, a downward trend in T_mean_ is observed across future periods for all RCP scenarios, followed by a subtle upward trend (Fig. 6a–c). On the other hand, in the SSP scenarios (Fig. 6j-l), there is a fluctuation in T_mean_ from no trend to an increasing trend across all three time periods. Similarly, at the Bandar Lengeh (Fig. 6d–f) and Bandar Abbas (Fig. 6g–i)) stations in the western and central parts respectively, the T_mean_ changes during the three time periods under the RCPs show fluctuations, including an increase, no trend, and finally a decrease. In contrast, under the SSPs, there is a clear increasing trend in T_mean_ for all three time periods across the region (Fig. 6m-r). Overall, the T_mean_ changes in the flat areas of the region show a consistent pattern, while there are some variations when examining specific stations and different scenarios (Fig. 6).

**Fig. 7:**
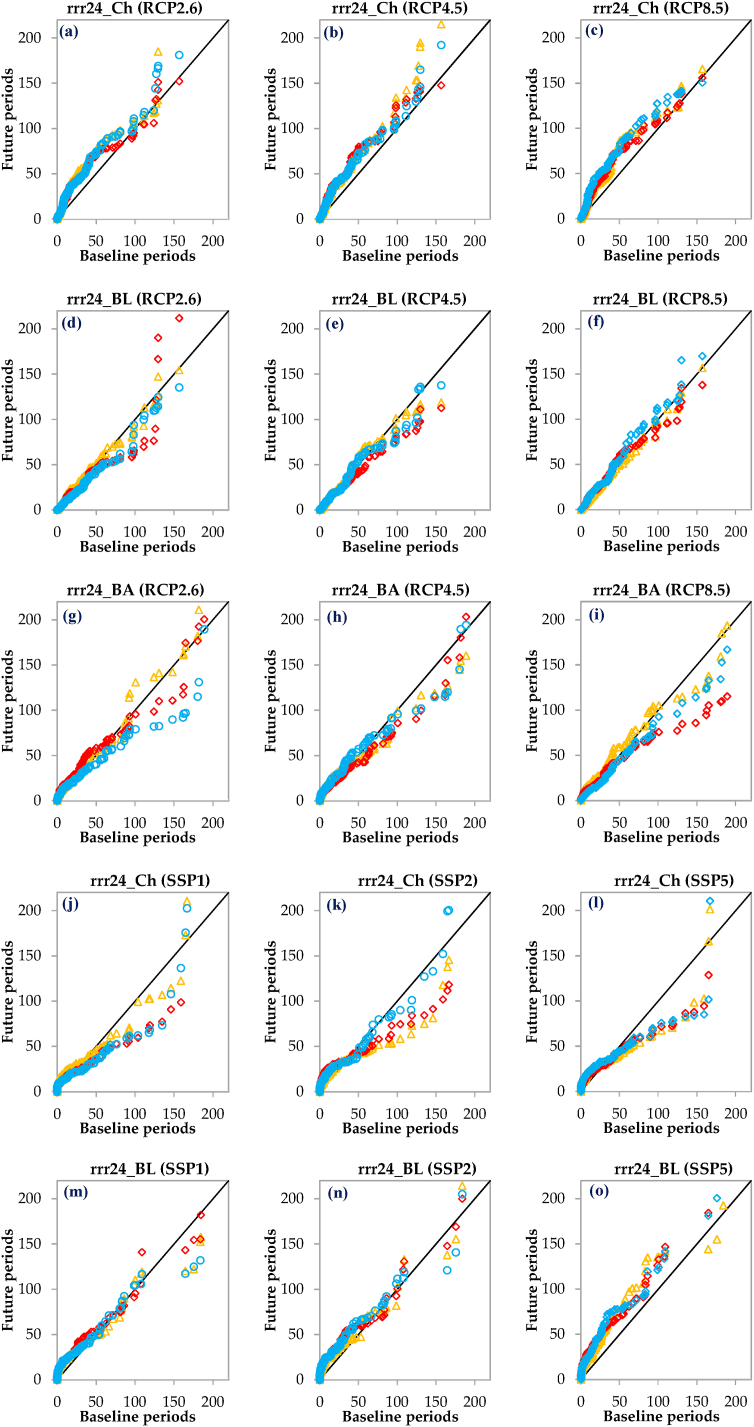

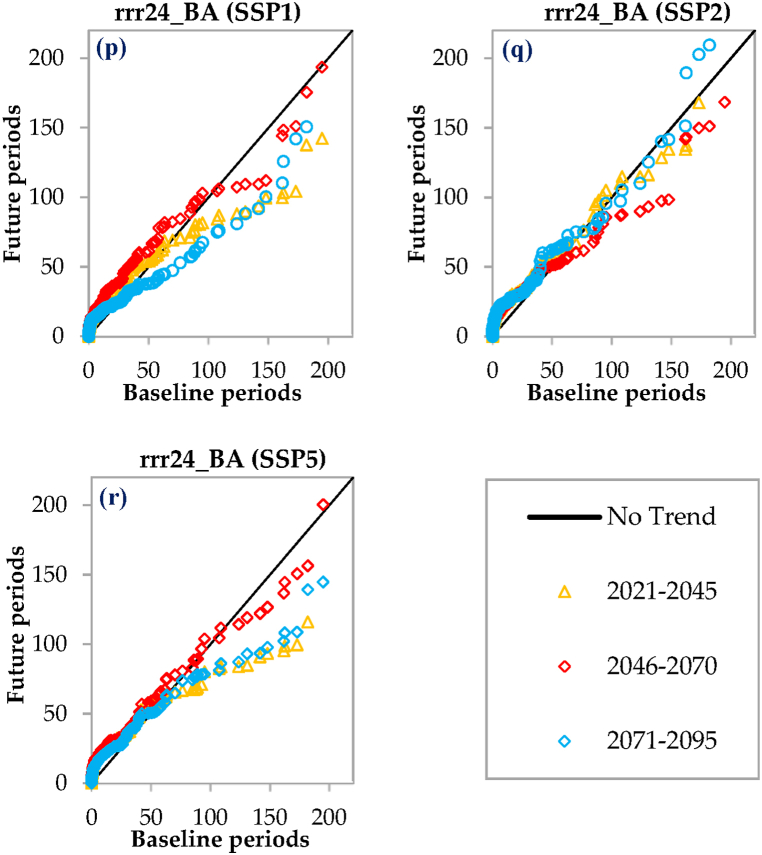
Innovative Trend Analysis (ITA) plots for comparing the 2021–2045, 2046–2070, and 2071–2095 periods to the 1980–2009 across various scenarios. rrr24 represents the 24 h accumulated rainfall, while Ch, BL, and BA represent the Chabahar, Bandar Lengeh, and Bandar Abbas stations, respectively.

As depicted in Fig. 7, the variations in rrr24 across all examined scenarios and three stations demonstrate similar behavior in the initial years of each time period. However, as time elapses, the patterns of rrr24 changes diverge. In the eastern part of the region (Chabahar station), rrr24 changes during all three time periods and under all RCPs show an increasing trend, particularly in the period of 2021–2045, with the RCP4.5 scenario displaying the highest increase (Fig. 7a–c). On the other hand, the rrr24 changes under the SSPs in all three time periods exhibit fluctuations (Fig. 7j-l), including periods of no trend, increase, and decrease. Notably, there is an increase again in the periods of 2021–2045 and 2071–2095. In the western part of the region (Bandar Lengeh station), rrr24 changes during all three time periods under the RCPs display fluctuations without a clear trend, involving periods of decrease and increase (Fig. 7d–f). The RCP2.6 scenario, particularly in the period of 2046–2070, is associated with a more pronounced increase (Fig. 7d). However, the rrr24 changes during all three time periods under the SSP1 scenario (Fig. 7m) show no trend initially, followed by a decrease. Under the SSP2 scenario (Fig. 7n), there is an increase, followed by a decrease and then an increase again. Lastly, under the SSP5 scenario (Fig. 7o), there is a consistent increasing trend in rrr24 across all three time periods. Overall, the behavior of rrr24 changes varies between different time periods and scenarios, with fluctuations observed in some cases and clear trends in others. The regions located in the east and west of the area demonstrate notable differences in rrr24 changes across various scenarios and time periods. At the Bandar Abbas station, located in the central part of the region, the rrr24 changes exhibit different patterns under various scenarios and time periods (Fig. 7g–i and p–r). Under the RCP4.5 scenario, the rrr24 changes during all three time periods show fluctuations without a clear trend initially, followed by a decrease, and then an increase. Similarly, under the RCP8.5 scenario, the changes display no trend initially, followed by a decrease, and an incremental increase from 2021 to 2045. For the RCP2.6 scenario, the rrr24 changes during all three time periods initially show no trend, followed by a decrease. However, for the period of 2021–2045, there will be an increase, followed by a period without a clear trend, and then another increase in the period of 2071–2095. Under the SSP1 scenario, the rrr24 changes exhibit an increasing trend initially, followed by a period without a clear trend, a decrease, and only from 2046 to 2070, there will be another increase. In the case of SSP2 and SSP5 scenarios, the rrr24 changes show an increasing trend initially, followed by a period without a clear trend. From 2071 through 2095, there will be an increase, and for the periods of 2021–2045 and 2046–2070, there will be a decrease. Overall, the rrr24 changes at the Bandar Abbas station vary across different scenarios and time periods, with fluctuations, decreases, and increases observed in different combinations (Fig. 7g–i and p–r).

Thus, a significant increase in T_mean_ was observed across all the scenarios and time periods, with the most pronounced trend occurring in the middle and late 21st century future periods. This increase was already evident during the base period of 2021–2045 across all scenarios. Moreover, the fluctuations in precipitation throughout the region and across all scenarios were significant in the three examined future periods. The study reveals that the coastal area of Hormozgan province will experience rising temperatures and changing rainfall patterns in the future. These changes may lead to challenges such as increased water and energy consumption, the heightened risk of droughts or floods, and potential damage to agriculture and infrastructure. Also, over the past century global climate change has been observed which has had an impact on regional water resources through changes in precipitation, temperature and energy balance [65,66]. The water-dependent ecosystems are exposed to the risk of climate change through altered evaporation and precipitation. The hydrological reaction to climate change and warming includes changes in water level and flow [67]. As a result, change in climate has significant and drastic impacts on agriculture, environment, economy and the society. The visible disastrous effects of this global threat are on malnutrition, poor health, food crisis and decreased labor productivity [68]. Noted that climate projections by global climate models (GCMs) are subject to multi-source and considerable uncertainties. For instance, with comparing of CMIP5 and CMIP6, Zhang and Chen [69] reported that the uncertainty for temperature was higher over land areas than oceans, and higher in the Northern Hemisphere than the Southern Hemisphere. The uncertainty for precipitation was lower in midlatitudes and parts of the equatorial region, but higher in low latitudes and the polar region.

### 3.3 Time series analysis

**Fig. 8:**
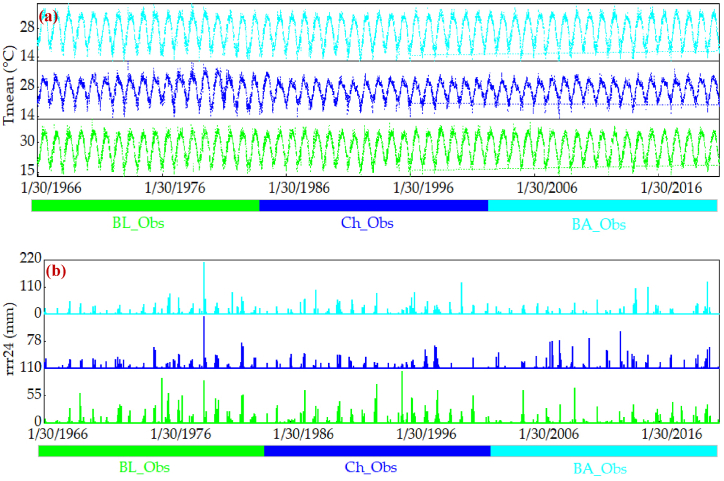
Time series of observed data of mean air temperature (panel a, T_mean_) and 24 h accumulated rainfall (panel b, rrr24) for 1966–2020 in 3 meteorological stations. BA, BL, and Ch denote the Bandar Abbas, Bandar Lengeh, and Chabahar stations, respectively.

**Fig. 9:**
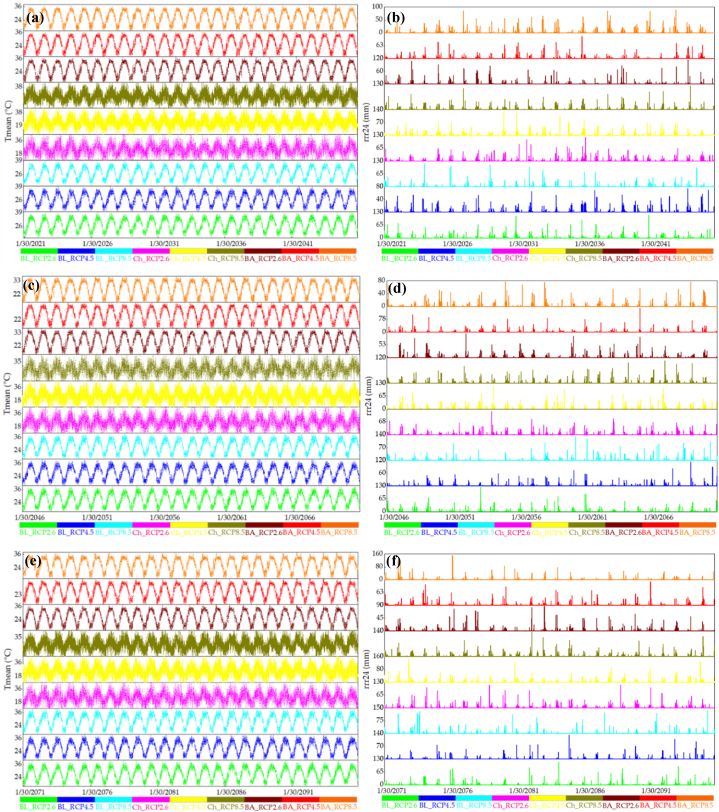
Time series of modeled future data of mean air temperature (T_mean_) and 24 h accumulated rainfall (rrr24) for 2021–2045 (panels a and b), 2046–2070 (panels c and d), and 2071–2095 (panels e and f) periods under RCP2.6, RCP4.5, and RCP8.5 scenarios in CanESM2 and CMIP5 model in 3 meteorological stations. BA, BL, and Ch denote the Bandar Abbas, Bandar Lengeh, and Chabahar stations, respectively.

**Fig. 10:**
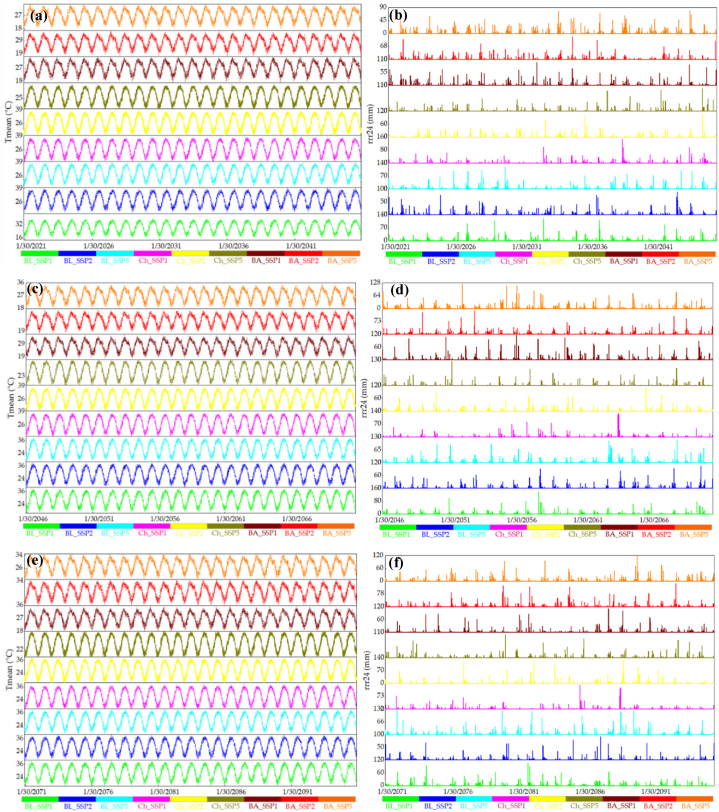
Time series of modeled future data of mean air temperature (T_mean_) and 24 h accumulated rainfall (rrr24) for 2021–2045 (panels a and b), 2046–2070 (panels c and d), and 2071–2095 (panels e and f) periods under SSP1, SSP2, and SSP5 scenarios in CanESM5, and CMIP6 models in 3 meteorological stations. rrr24 represents the 24 h accumulated rainfall, while BA, BL, and Ch denote the Bandar Abbas, Bandar Lengeh, and Chabahar stations, respectively.

Fig. 8, Fig. 9, Fig. 10 present time series analyses for T_mean_ and rrr24 at three meteorological stations, covering historical data (1966–2020) and projections from CMIP5 and CMIP6 models for 2021–2045, 2046–2070, and 2071–2095. Fig. 8a demonstrates cyclic T_mean_ patterns along the coastal strip of Hormozgan province during 1966–2020, accompanied by repetitive rrr24 fluctuations (Fig. 8b). Bandar Abbas and Chabahar stations show rising rrr24 trends in the late 1970s, while Bandar Lenge station exhibits increases in the 1970s and 1990s (Fig. 8b). Fig. 9 displays modeled future T_mean_ data under RCP2.6, RCP4.5, and RCP8.5 for 2021–2095, suggesting a consistent intermittent trend. This trend persists in Fig. 9 for the same periods, confirming cyclic patterns in projected T_mean_ data using CanESM2 and CMIP5 models (Fig. 9a–c and e). Fig. 9 offers insights into projected rrr24 data at the Bandar Abbas station for 2021–2045 and 2046–2070, influenced by CanESM2 and CMIP5 models (Fig. 9b–d, and f). Projections reveal cyclic changes across scenarios, slower in RCP4.5 and RCP2.6 compared to RCP8.5. These cyclic changes appear less pronounced during 2071–2095 for RCP2.6, RCP4.5, and RCP8.5, compared to prior periods. Notably, precipitation changes depicted by RCP2.6, RCP4.5, and RCP8.5 scenarios are less prominent during 2071–2095. Particularly, RCP8.5 projects have lower precipitation than earlier periods. Chabahar station shows cyclic changes for 2021–2045 across scenarios, milder in RCP4.5. This trend continues during 2046–2070 for all scenarios but less noticeably during 2071–2095 in RCP4.5 and RCP8.5. Similar cyclic changes emerge in the Bandar Lengeh station for 2021–2045 across scenarios. During 2046–2070, RCP4.5 and RCP8.5 exhibit reduced cyclic changes, with RCP4.5's rate reduction evident in 2071–2095. Fig. 10 analyzes future T_mean_ data for 2021–2045, 2046–2070, and 2071–2095 under SSP1, SSP2, and SSP5 using CanESM5 and CMIP6 models at three meteorological stations. This figure indicates a continuation of intermittent historical temperature trends. Furthermore, Fig. 10 (panels a to f) showcases projected rrr24 data for Bandar Abbas station during 2021–2045 and 2071–2095. Cyclic changes occur across scenarios, with reduced cycling in SSP2 and SSP5 during 2046–2070. Notably, these cyclic changes persist in Chabahar and Bandar Lengeh stations across scenarios and time periods.

Table 2 shows the results of T_mean_ changes magnitudes under different scenarios and time periods. The results indicated that among CMIP5 scenarios, RCP8.5 had highest changes of T_mean_ (+1.22 °C) in Bandar Lengeh station in 2071–2095 period. The lowest change magnitude of T_mean_ among CMIP5 scenarios was found in RCP4.5 (−1.94 °C) in Chabahar station in 2046–2070 period. Furthermore, the results indicated that among CMIP6 scenarios, SSP1 had highest changes of T_mean_ (+1.43 °C) in Bandar Lengeh station in 2021–2045 and 2071–2095 periods. The lowest change magnitude of T_mean_ among CMIP6 scenarios was found in SSP1 (+1.05 °C) in Bandar Abbas station in 2021–2045 period. In line with current study, Almazroui et al. [70] reported that temperature was projected to increase over the entire domain under all three SSPs, by as much as 6 °C under SSP5, and with more pronounced increases in the northern latitudes over the regions that receive snow in the present climate in United States, Central America and the Caribbean. Table 3 shows the results of rrr24 changes magnitudes under different scenarios and time periods. The results indicated that among CMIP5 scenarios, RCP8.5 had highest changes of rrr24 (+150.23 mm) in Chabahar station in 2071–2095 period. The lowest change magnitude of rrr24 among CMIP5 scenarios was found in RCP8.5 (−25.77 mm) in Bandar Abbas station in 2046–2070 period. Furthermore, the results indicated that among CMIP6 scenarios, SSP5 had highest changes of rrr24 (+85.69 mm) in Bandar Lengeh station in 2046–2070 period. The lowest change magnitude of rrr24 among CMIP6 scenarios was found in SSP2 (−9.12 mm) in Chabahar station in 2021–2045 period. Also, the results indicated that rrr24 was increasing in the most used CMIP5 and CMIP6 scenarios. Kamruzzaman et al. [71] reported that in the far future (2075–2100), the anticipated average annual precipitation increased by 9.48 %, 13.63 %, 21.07 %, and 30.90 % across Bangladesh.

**Table 2:** Mean air temperature (T_mean_, °C) changes magnitudes under different scenarios and time periods.

Station	Periods	Climate change scenario					
		RCP2.6	RCP4.5	RCP8.5	SSP1	SSP2	SSP5
Bandar Lengeh	2021–2045	0.03	−0.01	0.14	1.43	1.40	1.41
	2046–2070	0.11	0.36	0.69	1.43	1.42	1.41
	2071–2095	0.18	0.49	1.22	1.40	1.42	1.41
Bandar Abbas	2021–2045	0.18	0.15	0.20	1.05	1.08	1.07
	2046–2070	0.21	0.31	0.52	1.09	1.09	1.06
	2071–2095	0.23	0.36	0.74	1.08	1.06	1.09
Chabahar	2021–2045	−1.84	−1.94	−1.87	1.14	1.15	1.15
	2046–2070	−1.78	−1.47	−1.12	1.12	1.14	1.13
	2071–2095	−1.83	−1.34	−0.51	1.15	1.14	1.14

**Table 3:** The 24 h accumulated rainfall (rrr24, mm) changes magnitudes under different scenarios and time periods.

Station	Periods	Climate change scenario					
		RCP2.6	RCP4.5	RCP8.5	SSP1	SSP2	SSP5
Bandar Lengeh	2021–2045	47.36	46.67	50.01	35.41	39.36	71.58
	2046–2070	33.18	20.24	54.18	42.41	49.01	85.69
	2071–2095	18.97	41.29	67.32	35.13	42.88	80.16
Bandar Abbas	2021–2045	24.63	6.45	23.60	23.50	42.88	28.13
	2046–2070	21.01	−9.54	−25.77	60.18	21.32	35.29
	2071–2095	−21.33	13.52	−25.44	−8.00	56.54	11.11
Chabahar	2021–2045	73.41	136.41	127.13	39.31	−9.12	7.91
	2046–2070	67.21	132.56	120.29	16.89	8.49	−0.18
	2071–2095	65.44	124.27	150.23	4.50	19.92	11.20

### 3.4 Research limitations and future directions

The geographical region under investigation is characterized by cross-border disputes and possesses a restricted quantity of weather stations, thereby impacting the precision of climatic projections. It is suggested that, in future studies, a more accurate evaluation of the climatic changes in this region can be achieved by incorporating additional scenarios from both CMIP5 and CMIP6. Undoubtedly, such an approach will facilitate a more comprehensive investigation into the climate changes occurring in the region. Furthermore, the utilization of other CMIP5 models, such as MPI-ESM-MR, CSIRO-MK-3-6-0, and CMCC-CESM, is also suggested for inclusion in upcoming studies. Given the uncertainty surrounding climate change projections and the potential divergence of future climate patterns, implementing suitable precautionary measures can mitigate ecological, social, and economic losses. T_mean_ and rrr24 are the primary climate parameters extensively employed in evaluating the impacts of climate change. However, an inclusive analysis necessitates the incorporation of additional climate elements (e.g., wind speed), alongside T_mean_ and rrr24, to study factors like reference evapotranspiration. The alterations these variables undergo due to climate change remain less explored. Consequently, evaluating the capability of CMIP5 and CMIP6 models to accurately reproduce these variables holds significance. Certainly, for a comprehensive grasp of climate changes within this region in the future, it is imperative to undertake more intricate investigations. This entails accounting for natural climate influencers, including shifting oceanic conditions, such as oceanic modes of variability like the Indian Ocean Dipole [72]. This approach is crucial to acquire a more precise understanding and strategic planning.

## 4 Conclusions

The evaluation of model accuracy revealed that the CMIP5 model outperformed the CMIP6 model in simulating and predicting T_mean_ and rrr24, across all time periods and scenarios, this confirms the weakness of the CMIP6 model [25,31]. The climate projections by global climate models (GCMs) are subject to multi-source and considerable uncertainties*.* The CMIP6 models show weaknesses in capturing the climate characteristics in current study as a coastal area, particularly with overestimation in magnitudes. Thus, it is imperative to employ bias correction approaches in climate change projection studies by using CMIP6 outputs. There was a notable increase in T_mean_ observed in the coastal strip of Hormozgan province, specifically in Bandar Lengeh and Bandar Abbas stations. This increase was evident during the early 21st century (2021–2045) across all scenarios, with a more pronounced trend in the long-term future periods of 2046–2070 and 2071–2095. Among CMIP5 scenarios, RCP8.5 had highest changes of T_mean_ (+1.22 °C) in Bandar Lengeh station in 2071–2095 period. The lowest change magnitude of T_mean_ among CMIP5 scenarios was found in RCP4.5 (−1.94 °C) in Chabahar station in 2046–2070 period. Furthermore, in CMIP6 scenarios, SSP1 had highest changes of T_mean_ (+1.43 °C) in Bandar Lengeh station in 2021–2045 and 2071–2095 periods. Moreover, noteworthy precipitation fluctuations were observed across the region in all scenarios during the three future time periods. These significant variations underscore substantial variability in precipitation patterns. According to the IPCC report [65,66], there are no trends in drought and floods globally apparent in observed data yet. Due to the complexity of the hydroclimate, it could well be that some regions may see fewer droughts and floods than before, as rainfall belts may partly simply shift from one region to another. These conditions may pose various challenges, including heightened water and energy consumption, droughts or floods, and damage to agricultural lands and human infrastructure. The water-dependent ecosystems are exposed to the risk of climate change through altered evaporation and precipitation. The hydrological reaction to precipitation shifts includes changes in water level and flow. As a conclusion, change in variability in precipitation patterns has significant and drastic impacts on agriculture, forest ecosystems, economy and the society. These results hold significant implications as they can serve as a foundation for implementing practical mitigation policies and strategies on a local scale, as well as developing effective measures to adapt to these emerging climate changes. By doing so, we can work towards mitigating the adverse effects and enhancing the resilience of the coastal areas in Hormozgan province. These findings help managers to adopt local mitigation strategies and adaptive measures to decrease temperature and precipitation changes impacts in the study area. For example, using water harvesting technologies, watershed management practices, and new irrigation systems can be address the challenges like water consumption, agricultural impacts, and infrastructure vulnerability.

## Funding

This research received no external funding.

## CRediT authorship contribution statement

**Sorour Esfandeh:** Writing – original draft, Formal analysis, Data curation, Conceptualization. **Afshin Danehkar:** Supervision, Conceptualization. **Abdolrassoul Salmanmahiny:** Supervision, Conceptualization. **Hassan Alipour:** Validation, Software, Methodology, Formal analysis. **Majid Kazemzadeh:** Writing – review & editing. **Marina Viorela Marcu:** Writing – review & editing. **Seyed Mohammad Moein Sadeghi:** Writing – review & editing, Supervision, Project administration.

## Declaration of competing interest

The authors declare that they have no known competing financial interests or personal relationships that could have appeared to influence the work reported in this paper.

## Abbreviations list

GHG: Greenhouse gas
CMIP: Coupled Model Intercomparison Project
RCP: Representative Concentration Pathway
SSP: Shared Socioeconomic Pathway
T_mean_: Mean air temperature
rrr24: Total daily rainfall
NCEP: National Centre for Environmental Prediction
CanESM2: The Canadian Earth System Model version 2
CanESM5: The Canadian Earth System Model version 5
SDSM: Statistical Downscaling Model
MLR: Multi Linear Regression
SWG: Stochastic Weather Generator
GCM: General Circulation Model
OLS: Ordinary Least Squares
MSE: Mean square error
RMSE: Root mean square error
MAD: Median absolute deviation
ITA: Innovative- Sen Trend Analysis
BA: Bandar Abbas
BL: Bandar Lengeh
Ch: Chabahar
